# Prospects of formamide as nitrogen source in biotechnological production processes

**DOI:** 10.1007/s00253-023-12962-x

**Published:** 2024-01-10

**Authors:** Lynn S. Schwardmann, Leonie Benninghaus, Steffen N. Lindner, Volker F. Wendisch

**Affiliations:** 1 Genetics of Prokaryotes, Faculty of Biology and CeBiTec, Bielefeld University, Universitätsstr. 25, 33615 Bielefeld, Germany; 2 Aminoverse B.V., Daelderweg 9, 6361 HK, Nuth, Beekdaelen, The Netherlands; 3 Department of Biochemistry, Charite Universitatsmedizin Berlin, corporate member of Freie Universität Berlin and Humboldt-Universität, Berlin, Germany

**Keywords:** Formamide, Formamidase, Nitrogen source, C1 carbon source, Non-sterile fermentation, Formate

## Abstract

**Abstract:**

This review presents an analysis of formamide, focussing on its occurrence in nature, its functional roles, and its promising applications in the context of the bioeconomy. We discuss the utilization of formamide as an innovative nitrogen source achieved through metabolic engineering. These approaches underscore formamide’s potential in supporting growth and production in biotechnological processes. Furthermore, our review illuminates formamide’s role as a nitrogen source capable of safeguarding cultivation systems against contamination in non-sterile conditions. This attribute adds an extra layer of practicality to its application, rendering it an attractive candidate for sustainable and resilient industrial practices. Additionally, the article unveils the versatility of formamide as a potential carbon source that could be combined with formate or CO_2_ assimilation pathways. However, its attributes, i.e., enriched nitrogen content and comparatively limited energy content, led to conclude that formamide is more suitable as a co-substrate and that its use as a sole source of carbon for biomass and bio-production is limited. Through our exploration of formamide’s properties and its applications, this review underscores the significance of formamide as valuable resource for a large spectrum of industrial applications.

**Key points:**

• *Formidases enable access to formamide as source of nitrogen, carbon, and energy*

• *The formamide/formamidase system supports non-sterile fermentation*

• *The nitrogen source formamide supports production of nitrogenous compounds*

**Supplementary Information:**

The online version contains supplementary material available at 10.1007/s00253-023-12962-x.

## **Formamide occurrence in nature**

Formamide, also known as methanamide, is the simplest naturally occurring (monocarboxylic acid) amide and the smallest molecule with a peptide bond. Its composition includes hydrogen, oxygen, carbon, and nitrogen atoms, which belong to the seven most prevalent elements of the universe (Heiserman 1991), where it ubiquitously occurs (Saladino et al. 2012b). It is present in interstellar clouds (Solomon 1973) and on comets (Despois et al. 2002), estimated to constitute 0.015% of cometary ice in relation to H_2_O. Furthermore, formamide is a common molecule in star-forming regions in the galactic habitable zone in dense molecular clouds (Adande et al. 2013) and was detected at the galactic center of the Milky Way (Rubin et al. 1971; Gottlieb et al. 1973). Databased hypotheses suggested the presence of liquid formamide in a stratosphere under the frozen mantle surface of celestial bodies of our solar system, including some of the largest icy moons such as Saturn’s satellite Titan (Parnell et al. 2006) and Jupiter’s satellite Europa (Levy et al. 2000; Borucki et al. 2002).

The role of formamide in prebiotic chemistry and the origin of life is controversially discussed (Saladino et al. 2012a). Formamide may have been a precursor for the synthesis of a broad variety of biogenic molecules such as nucleoside bases (Ferus et al. 2015), sugars (Saladino et al. 2015), carboxylic acids, or amino acids (Saladino et al. 2013), with energy provided in form of heat or UV radiation (Saladino et al. 2012b) in the presence of mineral catalysts (Bizzarri et al. 2021).

In some microorganisms, formamide occurs as a degradation product of histidine and cyanide (Wachsman and Barker 1955; Ferber et al. 1988; Kunz et al. 1994). Formamide is a rare metabolite in microbes, e.g., while its role in nitrogen metabolism of *Heliobacter pylori* as a nitrogen source as well as for protection at acidic conditions is clear, the source of formamide in its habitat, the human gastro-intestinal system, is currently unknown (Skouloubris et al. 2001).

Formamide has an annual market estimated to reach US $270 million by 2027 with the four major uses as feedstock for producing agrochemicals (pesticides, herbicides), plastics, paper, and card board or as solvent in plasticizers for making concrete (IndustyArc, 2022). Chemical synthesis of formamide proceeds by direct carbonylation of ammonia or by aminolysis of methyl formate, and both processes occur under high-temperature and high-pressure conditions. Very recently, electrochemical synthesis of formamide under ambient conditions has been described from either ammonia and methanol (Meng et al. 2022), ammonia and carbon dioxide (Li and Kornienko, 2022), and nitrate or ammonia, or carbon monoxide and nitrite (Lan et al. 2023) in a similar manner as electrosynthesis of methylamine from carbon dioxide and nitrate (Wu et al. 2021). Its uses comprise industrial production of hydrogen cyanide, its application as cryoprotectant or as ionizing solvent in aqueous buffers, but also molecular biology uses such as destabilizing double helices in RNA gel electrophoresis are established (Böckler et al. 2018).

## Formamide as nitrogen and/or carbon source for bacteria

**Fig. 1:**
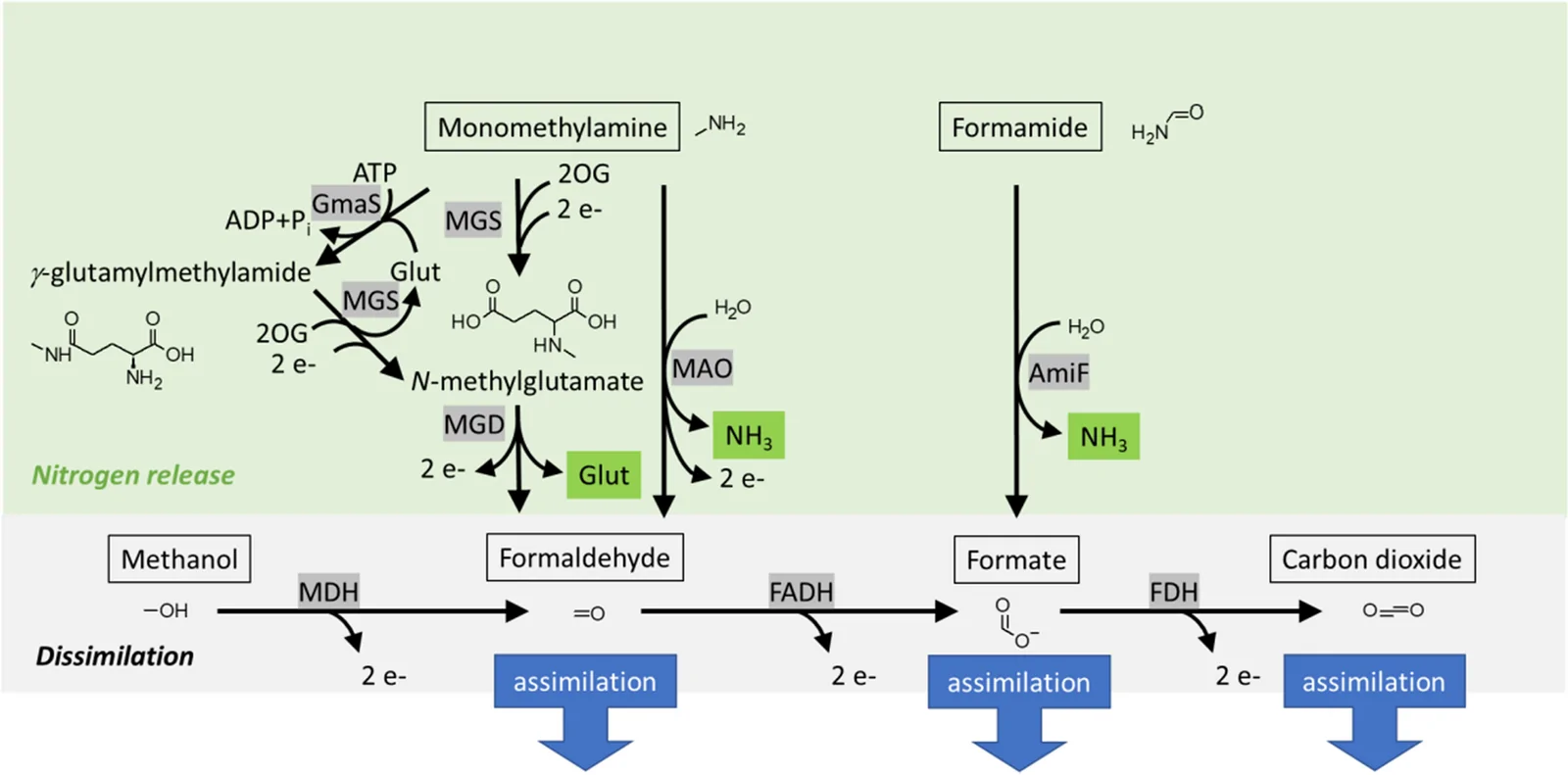
Catabolism of the reduced C1 nitrogen compounds monomethylamine and formamide. Enzymes are boxed in dark grey, liberation of nitrogen as either ammonia or L-glutamate is indicated by boxing in green, linear dissimilation of methanol to carbon dioxide is shaded in light grey, and assimilation of formaldehyde, formate, and carbon dioxide is indicated by blue arrows. Abbreviations: 2 e-, transfer of 2 electrons from various redox cofactors; 2OG, 2-oxoglutarate; AmiF, formamidase (EC 3.5.1.49); FADH, formaldehyde dehydrogenase; FDH, formate dehydrogenase; Glut, L-glutamate; GmaS, γ-glutamylmethylamide synthase (EC 6.3.4.12); MAO, monomethylamine oxidase (EC 1.4.9.1); MDH, methanol dehydrogenase, MGS, *N*-methylglutamate synthase (operating comparable to either glutamate dehydrogenase (EC 1.4.1.2/3/4) or glutamine oxoglutarate aminotransferase GOGAT (EC 1.4.1.13/14)); MGD, *N*-methyl-L-glutamate dehydrogenase (EC 1.5.99.5)

Formamide belongs to the reduced C1 nitrogen compounds. While ammonium carbamate and carbamoyl phosphate readily decompose to yield ammonia (also catalyzed by carbamate kinase; EC 2.7.2.2; Pols et al. 2021), the liberation of nitrogen from formamide and monomethylamine, a related C1 nitrogen source, occurs by enzyme catalyzed reactions (Fig. 1). In the case of formamide, the straightforward amide hydrolysis reaction catalyzed by formamidase (EC 3.5.1.49; AmiF/FmdA) yields ammonia and formate. In the case of monomethylamine, nitrogen can be liberated either as ammonia or as L-glutamate. Oxidative deamination of monomethylamine by monomethylamine oxidase (EC 1.4.9.1; MAO) in Gram-positive methylotrophs (Iersel et al. 1986; Dooley et al. 1990; Cai and Klinman 1994) or by a periplasmic methylamine dehydrogenase (Eady and Large 1968; Chistoserdov 1991) yields ammonia and formaldehyde. Alternatively, monomethylamine can first be converted to formaldehyde via *N*-methyl-L-glutamate in methylotrophs and non-methylotrophs (Chen et al. 2010). Two options for the synthesis of *N*-methyl-L-glutamate from monomethylamine and 2-oxoglutarate exist. On the one hand, *N*-methylglutamate synthase (MGS) reductively methylaminates 2-oxoglutarate to *N*-methyl-L-glutamate in a reaction comparable to glutamate dehydrogenase (GDH, EC 1.4.1.2/3/4). On the other hand, *N*-methyl-L-glutamate is formed in a two-step sequence involving γ-glutamylmethylamide synthase (GmaS, EC 6.3.4.12) for ATP-dependent methylamidation comparable to glutamine synthetases (GS) followed by MGS operating comparable to glutamine oxoglutarate aminotransferase GOGAT (EC 1.4.1.13/14) (Bamforth and O’Connor 1979). Thus, synthesis of *N*-methyl-L-glutamate from monomethylamine and 2-oxoglutarate is similar to the well-known GDH and GS/GOGAT reactions for L-glutamate synthesis from ammonia and 2-oxoglutarate (Mindt et al. 2018). Subsequently, *N*-methyl-L-glutamate is oxidized by *N*-methyl-L-glutamate dehydrogenase (EC 1.5.99.5, MGD) to yield formaldehyde and L-glutamate. L-glutamate is either oxidatively deaminated to 2-oxoglutarate and ammonia or can be used in transamination reactions directly.

While utilization of monomethylamine as C source may occur by assimilation of the generated formaldehyde (e.g., in the ribulosemonophosphate cycle or the serine cycle), formamide yields formate that can be assimilated or oxidized to carbon dioxide, which in turn may be assimilated (e.g., via the Calvin-Benson-Bassham cycle; see the “Formamide as potential C source in biotechnology” section). Efficient utilization of monomethylamine and formamide as nitrogen sources requires prompt dissimilation and/or assimilation of formaldehyde and formate to avoid growth inhibition, which is particularly relevant in the case of formaldehyde.

Since the first observation of formamide utilization in 1976 for *Pseudomonas* SL-4, which can utilize formamide as sole nitrogen, carbon, and energy source as part of an ecological carbon-nitrogen cycle (Thatcher and Weaver 1976), this trait has been found in other bacteria such as *Paracoccus aminophilus* and *Pseudomonas putida*. However, formamide utilization by bacteria is rare as compared to the abundance of bacteria utilizing ammonia, nitrite, and nitrate as nitrogen sources. Thus, transfer of this rare metabolic trait to industrially relevant bacteria offers unique application opportunities.

## Formamidase: phylogeny, enzyme activity, biochemical and genetic regulation

The enzyme sub-class EC 3.5.1 comprises many enzymes hydrolyzing linear C–N bonds other than peptide bonds. Some enzymes are active on *N*-formylated amino acids such as *N*-formyl-L-aspartate, *N*-formyl-L-methionine, *N*-methyl-anthranilate, *N*-formyl-L-kynurenine, or on 10-formyltetrahydrofolate. Formamidases (EC 3.5.1.49), also known as formamide aminohydrolases, catalyze the hydrolysis of the amide bond in formamide to release formate and ammonia.

Although amidases are widespread among bacteria for degradation of toxic amides (Newton et al. 2000; Fournand and Arnaud 2001; Liu et al. 2020), only few formamide-specific amidases have been identified. Formamidases were detected in *Helicobacter pylori* (Wyborn et al. 1996; Skouloubris et al. 2001), *Bacillus cereus* (Soriano-Maldonado et al. 2011), *Streptomyces parvulus* (Brown et al. 1986), *Methylophilus methylotrophus* (Wyborn et al. 1994), *Cupriavidus necator* (formerly known as *Ralstonia eutropha*) (Friedrich and Mitrenga 1981), and *Sinorhizobium meliloti* (Yurgel et al. 2022), but only some of these have been structurally and biochemically characterized. Beyond that, formamidases are found in fungi like *Aspergillus nidulans* (Hynes 1975; Fraser et al. 2001) or *Paracoccidioides brasiliensis* (Borges et al. 2005) and plants like *Arabidopsis thaliana* (Fraser et al. 2001) or in the roots of white lupin (*Lupinus albus* L.) (Rath et al. 2010). Their unambiguous classification is complicated by the ability of other enzymes such as acetamidase from *Mycobacterium smegmatis* (Draper 1967) and some aliphatic amidases (Egorova et al. 2004; Makhongela et al. 2007; Engelhardt et al. 2009) to hydrolyze formamide and led to the incorrect annotation of > 20 proteins (Soriano-Maldonado et al. 2011).

**Fig. 2:**
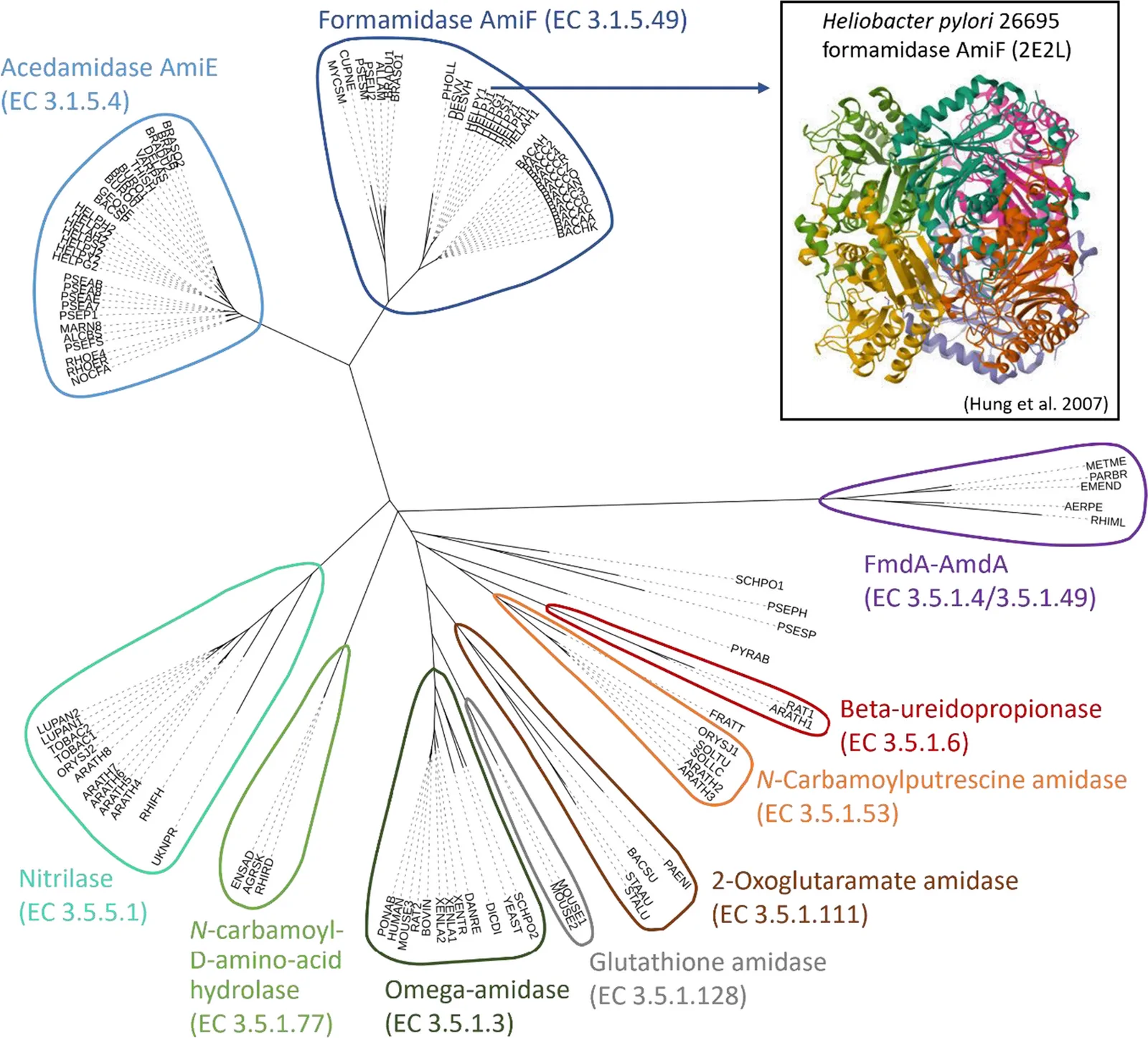
Phylogenetic tree of formamidase AmiF (*H. pylori* 26695) homologues depicted with the protein structure of AmiF of *H. pylori*. The tree includes the 100 most homologous proteins to formamidase AmiF from *H. pylori* 26695 (HELPY), identified by PSI-BLAST, and 7 further formamidase proteins of biotechnological interest. The identified enzymes are assigned to at least 9 different enzyme classes (outlined) and some lack classification. Labels refer to the host organism, as defined in Tab. S1. The crystal structure 2E2L of AmiF from *H. pylori* (Hung et al. 2007) is depicted

Phylogenetic analysis revealed that formamidases belong to at least two different groups of enzymes, namely the acetamidase/formamidase super family (FmdA-AmdA, Pfam PF03069), which also includes amidohydrolases of acetamide (Draper 1967), and the nitrilase family, which is a subfamily of the carbon-hydrogen nitrilase superfamily and hydrolyses various nitriles, producing ammonia and the respective carboxylic acid (Bessonnet et al. 2021; Teepakorn et al. 2021). Although both have certain sequence similarities with members of the major amidase families like aliphatic amidases, acylamide aminohydrolases, and nitrilase/cyanide hydratases, these two groups are demarcated (Fig. 2). Between each other, they possess less than 10% sequence similarity (Soriano-Maldonado et al. 2011) and differ in their molecular masses of around 45 kDa (FmdA-AmdA) (Wyborn et al. 1994; Wyborn et al. 1996; Borges et al. 2010) and 34 kDa (nitrilases) (Skouloubris et al. 2001; Thuku et al. 2009; Soriano-Maldonado et al. 2011), respectively. The low similarity between the amino acid sequences of the formamidases of the Fmda-AmdA superfamily member *Mycobacterium smegmatis* and the nitrilase superfamily member *Methylophilus methylotrophus* led to the hypothesis of their evolutionary emergence from a common ancestral protein or by early horizontal gene transfer (Wyborn et al. 1996). *H. pylori* formamidase AmiF (EC 3.5.1.49) was discovered as putative paralogue to aliphatic amidase AmiE of *H. pylori* and was the first described formamidase of the nitrilase family. AmiF and AmiE share 34% of the amino acid sequence but differ in substrate specificity. AmiF activity is restricted to formamide, whereas AmiE possesses a broader substrate spectrum and was demonstrated to act on propionamide, acetamide, and acrylamide (Skouloubris et al. 2001). Thus, AmiE and AmiF of *H. Pylori* have supposedly evolved after ancestral gene duplication and represent specialized paralogues (Skouloubris et al. 2001).

Homology database searches using the FmdA-AmdA superfamily formamidase sequence of *A. nidulans* found that highly conserved formamidase-like sequences are not restricted to microorganisms but also distributed among eukaryotes like *A. thaliana* and archaea like *Aeropyrum pernix* (Fig. 2)*.* Functional formamidase expression was exemplarily verified for *A. thaliana* and the fission yeast *Schizosaccharomyces pombe* (Fraser et al. 2001).

While the enzyme activities of some formamidases have been characterized in fair detail, a systematic analysis of the substrate specificity beyond formamide is absent. Among the characterized formamidases of the nitrilase family, none acted on urea. However, while *H. pylori* formamidase only showed activity with formamide but not with acetamide, acrylamide, or propionamide (Skouloubris et al. 2001), *M. methylotrophus* formamidase hydrolyzed acetamide, butyramide, propionamide, and acrylamide (Wyborn et al. 1996), and *B. cereus* formamidase also accepted acetamide besides formamide but not alaninamide, butyramide, isobutyramide, leucinamide, glycinamide, or propionamide (Soriano-Maldonado et al. 2011). These divergences are reflected in the kinetic parameters of these enzymes as summarized in Table 1. From a thermodynamic perspective, the formamide cleavage direction is highly favored over the reverse reaction (Beber et al. 2022). Additionally, formamidase’s specific enzyme activities (as shown in Table 1) are in the range of the activities of the average central metabolism enzyme (e.g., *kcat* of 64 s^−1^ for *M. methylotrophus* Wyborn et al. 1994) (Soares et al. 2011) and, most importantly, exceed the activities reported for glutamate dehydrogenase (GDH), the primary NH_3_ assimilation reaction (e.g., 0.18 mmol min^−1^ mg^−1^ of GDH of *E. coli* Sakamoto et al. 1975).

**Table 1:** Experimentally determined kinetic parameters of formamidases from *A. nidulans*, *B. cereus*, *H. pylori*, *L. albus,* and *M. methylotrophus* with formamide. n.d., not determined

Organism	Family	*K*_*m*_ (mM)	*Specific activity* (mmol min^−1^ mg^−1^)	Assayed conditions	Conditions for maximal activity	References
*B. cereus*	Nitrilase	108 ± 17	5.8 ± 0.4	50 °C, pH 6, purified enzyme	50 °C, pH 6	(Soriano-Maldonado et al. 2011)
*H. pylori*	Nitrilase	32 ± 9	1.11 ± 0.1	30 °C, pH 7.4, purified enzyme	45 °C, pH 6	(Skouloubris et al. 2001)
*L. albus*	FmdA-AmdA	71 ± 15	n.d.	30 °C, pH 7.4, protein crude extract from proteoid roots of nitrogen-deprived white lupin	35–45 °C, pH 6–8	(Rath et al. 2010)
*M. methylotrophus*	FmdA-AmdA	2.1	0.037	Purified enzyme	37 °C, pH 6	(Wyborn et al. 1994)
*M. smegmatis*	Putatively FmdA-AmdA, originally assigned as acetamidases	n.d.	0.17	37 °C, pH 7.2 acetamide as carbon source, partially purified enzyme preparation	pH 8-9	(Draper 1967)

AmiF of *H. pylori* and *B. cereus* belong to the nitrilase superfamily. A characteristic of this family is the presence of a C-E-K (Cys-Glu-Lys) triad in which the active cysteine acts as the nucleophile, glutamate mediates the proton transfer, and lysine stabilizes the tetrahedral transition state (Hung et al. 2007). The hydroxylation of formamide by AmiF from *H. pylori* comprises two phases. (1) During the acylation reaction, formamide diffuses into the pocket and binds onto the C-E-K triad. Glu^60^ mediates the proton transfers from the –SH group of C166 to initiate the first attack to the carbon atom of formamide. The transition state negatively charged intermediate is formed, and the instability of the substrate carbonyl oxygen results in the collapse of this intermediate. This leads to the production of an acyl-enzyme intermediate, breaking the C–N bond, and the release of an NH_3_ molecule. (2) The acyl-enzyme intermediate is deacylated. Glu^60^ deprotonates a water molecule to start the second nucleophilic attack. Again, a tetrahedral intermediate is formed, the collapse of the unstable intermediate yields a formic acid molecule, and the enzyme is regenerated. In AmiF of *B. cereus*, W136 is essential for the conformational stability of the enzyme (Soriano-Maldonado et al. 2011), while D168 is the essential residue in AmiF of *H. pylori* (Skouloubris et al. 2001). The exchange of E140D prohibited enzymatic activity while the binding of formamide was not affected, indicating a key role of E140 for hydrolysis. For *H. pylori*, it was suggested that this amino acid residue maintains the side chain geometry of the catalytic C-E-K triad and facilitates the docking of the substrate. The amino acid residues Trp^137^ and Tyr^192^ generate an exterior wall and therefore form a small room at the active site, which only allows formamide as substrate (Hung et al. 2007).

Biochemical regulation of formamidase activity is known. Compounds such as Hg^2+^ ions, iodoacetamide, or iodoacetate inhibit the formamidases of *B. cereus and H. pylori* as they react with a catalytic cysteine residue (Skouloubris et al. 2001; Soriano-Maldonado et al. 2011). Urea and thiourea, which resemble formamide, inhibit the formamidases of *B. cereus *and* M. methylotrophus* (Wyborn et al. 1996; Martínez-Rodríguez et al. 2019), and the presence of Ni^2+^ ions activates the *H. pylori* enzyme (Bury-Moné et al. 2004).

Genetic regulation of formamidase genes has been described in response to the substrate formamide, the product nitrogen, iron, or carbon source availability. The formamidase gene *fmdS* from *A. nidulans* is regulated by AreA-dependent nitrogen metabolite repression and contains multiple GATA sequences in the promoter region for AreA binding (Hynes 1972; Fraser et al. 2001). No induction by formamide was observed, while the response to nitrogen limitation was reduced when carbon was also limited as the activation by AreA is lost (Hynes 1972; Fraser et al. 2001). Bacterial amidases are often induced by the presence of their amide substrates. For example, in *M. methylotrophus*, the gene cluster *fmdCABDEF*, which encodes a formamidase, a putative positive regulator, an outer-membrane porin for short-chain amides and urea, and the three subunits for binding protein-dependent high-affinity uptake of short-chain amides and urea (Wyborn et al. 1996), is induced by formamide and urea and repressed by high concentrations of ammonia (Mills et al. 1998). In the gastric pathogen *H. pylori*, *amiF* is not substrate-inducible, but the genes *amiE*, *amiF*, and *ureA* are transcriptionally upregulated by acid exposure (Merrell et al. 2003; Bury-Moné et al. 2004). Two metal-dependent transcriptional regulators, nickel homeostasis activator NikR and ferric uptake repressor FurR, are directly or indirectly involved in the acid induction of urease, amidase, and formamidase genes, although both amidases do not contain metal ions. At acidic pH, *amiF* is derepressed by Fur. Fur is epistatic on NikR, which represses *fur*. NikR directly responds to changes in cytosolic pH during acid acclimation as it shows pH-dependent DNA binding to its target promoter sequences (Jones et al. 2018). In addition, *amiF* is controlled by a yet unknown third regulator (Bury-Moné et al. 2004).

## Formamide for contamination-free, non-sterile cultivation

Microbial contamination constitutes a major obstacle to the stable performance of bioprocesses, may hamper their economically competitive implementation, and is a threat to product quality and safety (Neu 1992). Typically, this risk is encountered by the addition of antimicrobial agents and sterilization of fermentation vessels, laboratory equipment, and cultivation media (Guo et al. 2020b). However, these measures are expensive in cost, resources, energy, and time and favor the emergence of drug-resistant strains (Neu 1992; Guo et al. 2020b). Therefore, their replacement for innovative, more sustainable strategies is highly desirable.

Recently, some biofuels and chemicals have been successfully produced under non-sterile conditions by exploiting the capacity of extraordinary microbes to withstand inhospitable conditions or to assimilate uncommon macronutrient sources as a selective trait (Thorwall et al. 2020). Most successful approaches relied on the former, e.g., cultivation at elevated temperature of ≥ 50 °C enabled the non-sterile production of poly-γ-glutamate, acetoin, and lactic acid by thermophilic *Bacilli* strains (Zeng et al. 2013; Zhang et al. 2014; Xiao et al. 2017) and an evolved thermotolerant strain of *Thermoanaerobacterium aotearoense* (Yang et al. 2015). The salt and pH tolerance of *Halomonas bluephagenesis* and *Halomonas campaniensis* LS21 was exploited for the non-sterile production of poly-3-hydroxybutyrate-co-4-hydroxybutyrate (Ye et al. 2018) and poly-3-hydroxybutyrate (Jiang et al. 2017). The non-sterile production of an anticancer polysaccharide by an evolved methanol-tolerant mutant strain of *Chaetomium globosum* serves as another example (Wang et al. 2019). However, this strategy is limited to some extraordinary microbes as it is prone to slight environmental changes (Barig et al. 2011), and unwanted mutations may arise (Ling et al. 2014). Another limitation is that the desired product must tolerate the extraordinary culture conditions (Thorwall et al. 2020).

Alternatively, a competitive advantage can be conferred by engineering the target strain to harness a rare xenobiotic compound as an essential growth nutrient, which is not accessible to (most) competing microbes and thus enables cultivation and production in the respective auxotrophic medium. To construct an antimicrobial contamination system, naturally formamidase-deficient *B. subtilis* and *E. coli* were equipped with codon-optimized versions of formamidase genes from *H. pylori* 26695 and *Paenibacillus pasadenensis* CS0611, respectively. However, slight growth of the formamidase-deficient *E. coli* control strain with formamide was observed (Ou et al. 2019), which was in accordance with little growth of wild-type strains, when melamine or cyanamide assimilation was introduced as selective advantage (Shaw et al. 2016). Therefore, a second key nutritional constraint in the form of phosphite dehydrogenase-mediated phosphite utilization was added (Ou et al. 2019), yielding a dual protection system, which was similarly introduced in *B. subtilis* (Guo et al. 2020a). The power of the dual protection system was demonstrated by outcompeting representative eukaryotic and bacterial competitors since the engineered target strains constituted > 90% of the final culture composition after 30–35 h (Ou et al. 2019; Guo et al. 2020a). The metabolic selection pressure was sufficient to ensure the maintenance of the phosphite dehydrogenase-encoding plasmid for 17 serial dilutions, whereas the same plasmid was lost when commonly accessible phosphate was provided (Schwardmann et al. 2022), and enabled a non-sterile fed-batch fermentation for acetoin production (Guo et al. 2020a).

The presence of undesired competitor organisms was hypothesized to be due to leaked phosphite and formamide degradation products. This was demonstrated regarding ammonium leakage of ammonium from a formamidase-positive *C. glutamicum* strain that allowed growth of a second formamidase-deficient strain in co-cultivation (Schwardmann et al. 2023b). High formamidase activity (4-fold higher in crude extracts of *C. glutamicum* as compared to *B. subtilis*; 6 compared to 1.2 U mg^−1^) may have led to surplus ammonium formation (Guo et al. 2020a; Schwardmann et al. 2023b), indicating that the catalytic activity must be fine-tuned to the utilization capacity of the host to avoid nutrient leakage (Shaw et al. 2016).

Besides formamide, melamine and cyanamide present the only uncommon nitrogen sources exploited for contamination control in the cyanobacterium *Synechococcus* sp. PCC 7002 (Selão et al. 2019) and *E. coli*, *Saccharomyces cerevisiae*, and *Yarrowia lipolyptica*, respectively (Shaw et al. 2016), and shown to be applicable to prevent contamination (Shaw et al. 2016; Selão et al. 2019).

Taken together, the utilization of formamide as a rare xenobiotic nutrient provides a promising tool to ensure ample dominance of a formamidase-positive strain, although it cannot guarantee completely contamination-free non-sterile cultivation and necessitates appropriate expression levels.

## Formamide as potential C source in biotechnology

**Fig. 3:**
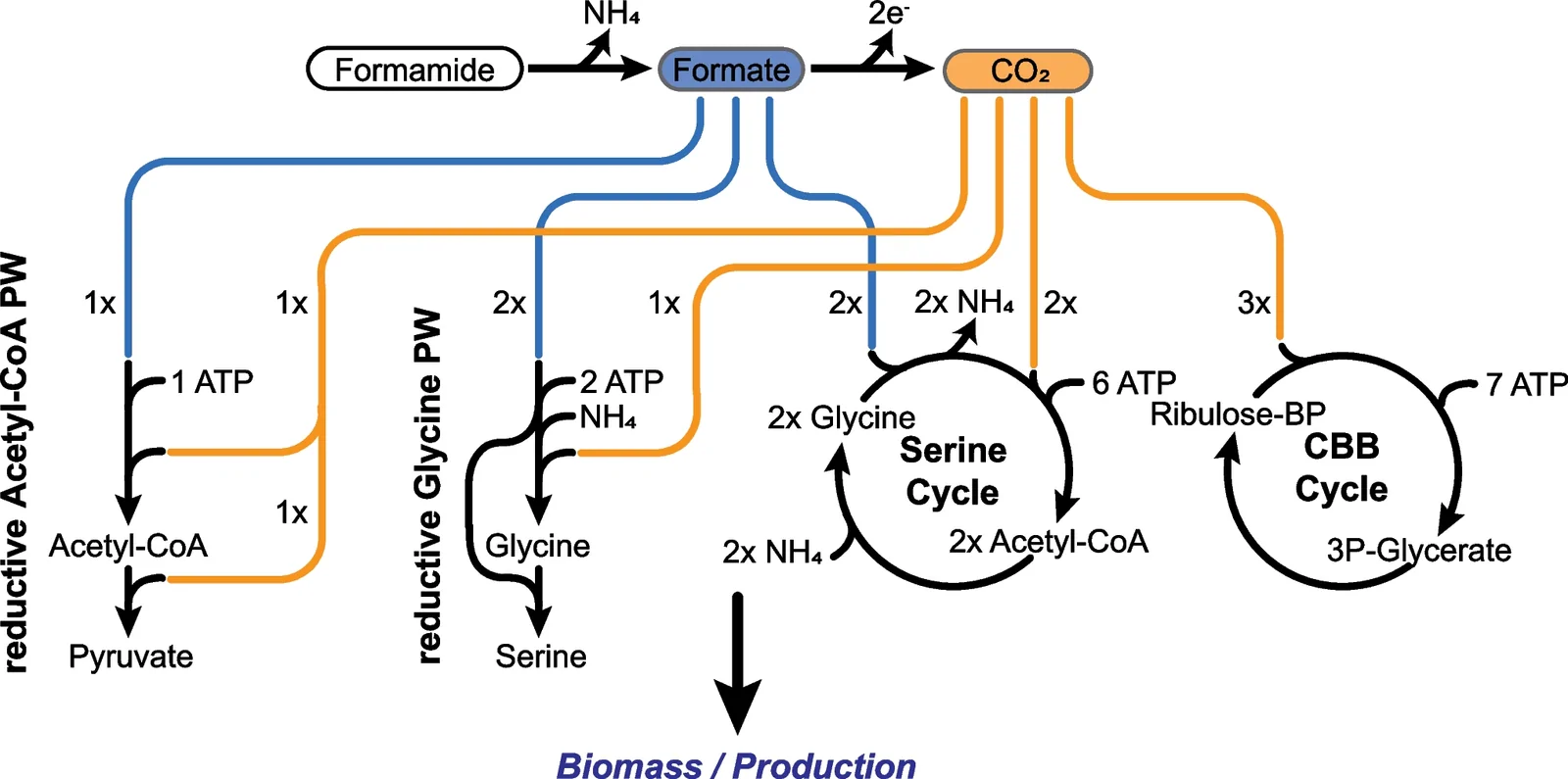
Formamide as a formate source to support growth and production via formate and CO_2_ assimilation pathway. Schemes illustrate formate (blue) and CO_2_ (orange) utilization via the reductive Acetyl-CoA pathway, the reductive Glycine pathway, the Serine Cycle, and the Calvin-Benson-Bassham (CBB) cycle (from left to right)

Formamide has the potential to serve as a source of carbon, nitrogen, and energy, supporting the growth of microorganisms for biotechnological applications. The enzymatic breakdown of formamide by formamidase yields formate, which is a natural carbon source and source of reducing power for certain formate-utilizing microorganisms. These organisms either utilize formate directly through pathways (Fig. 3) like the reductive acetyl-CoA pathway or the serine cycle or first oxidize formate to CO_2_ and use less efficient CO_2_ assimilation pathways such as the reductive pentose phosphate pathway (Calvin-Benson-Bassham cycle) (Bar-Even 2016). In recent years, the use of formate as a CO_2_-based renewable and scalable feedstock was proposed with the aim of promoting a sustainable bioeconomy (Yishai et al. 2016).

Consequently, there has been a concerted effort to introduce both natural and engineered routes for the incorporation of formate through direct formate or CO_2_ assimilation into biotechnologically significant bacteria and yeast (Fig. 3) (Yu and Liao 2018; Gleizer et al. 2019; Claassens et al. 2020; Kim et al. 2020; Turlin et al. 2022; Wenk et al. 2022; Bruinsma et al. 2023). These projects have led to the creation of synthetic autotrophs and formatotrophs. Here, particularly noteworthy is the successful implementation of the highly ATP-efficient synthetic reductive glycine pathway in *E. coli* (Claassens et al. 2020; Kim et al. 2020; Turlin et al. 2022; Bruinsma et al. 2023), which enables fast growth and high biomass yields of the engineered *E. coli* strains, outperforming the growth performance of some natural formatotrophic strains (Cotton et al. 2020; Kim et al. 2023). Nonetheless, a significant limitation of relying on formate as a sole feedstock arises from the necessity to oxidize a substantial portion of it to CO_2_ in order to generate the required reduction power to attain the reduction state of cellular biomass or the targeted bioproduct. This loss of feedstock for assimilation poses a challenge by influencing the carbon footprint and the resource efficiency of the process. To mitigate these shortcomings, CO_2_ recycling can contribute to enhance the sustainability of the process. In this context, feedstocks with higher levels of reduction, such as methanol, could potentially offer a more advantageous alternative.

When originating from formamide, a 1:1 proportion of formate to ammonium is produced. However, the elemental composition of a bacterial cell exhibits a carbon and nitrogen content of 4:1 (Milo and Phillips 2015). Thus, the equimolar provision of carbon and nitrogen by formamide is not ideal to support growth. By comparison, glucosamine represents a much more suitable sole source of carbon, nitrogen, and energy for *C. glutamicum* (Uhde et al. 2013). To address these limitations, an optimal strategy involves co-feeding formamide alongside supplementary carbon sources. This approach would result in reduced formate-derived CO_2_ loss and a more efficient utilization of the excess nitrogen inherent in formamide as a feedstock. Formate already has applications as a co-substrate, by either providing reducing power or by enhancing the carbon yield. For example, formate has been used as co-substrate together with glucose in order to balance reducing equivalents for anaerobic succinate production by *C. glutamicum* (Litsanov et al. 2012), as well as its co-substrate role with glucose in *Ustilago cynodontis* for itaconate production (Ullmann et al. 2022). Additionally, formate-derived CO_2_ was used to enhance ethanol production from glucose in *E. coli* (Tseng et al. 2018) and from glucose and galactose in *S. cerevisiae* (Guadalupe-Medina et al. 2013)*.*

However, some organisms do not easily support carbon source co-utilization due to metabolite repression mechanisms that lead to sequential carbon source utilization. This behavior is observed in bacteria such as *E. coli* (Alva et al. 2020) and *B. subtilis* (Fujita 2009)*.* Overcoming this limitation necessitates metabolic engineering strategies (Wendisch et al. 2016). Consequently, organisms that effectively co-utilize diverse carbon sources, e.g., *C. glutamicum* (Blombach and Seibold 2010; Teramoto et al. 2011), provide a clear process development advantage.

Taken together, while the use of formamide as nitrogen source is relevant for biotechnology applications, using formamide as sole carbon source is of limited value if the surplus of energy-intensive ammonium is not utilized. Here, supplementation of additional formate or other C1-compounds could be of value, similar co-feeding strategies with, e.g., sugar-based feedstocks.

## Formamide as N source in biotechnology

Biotechnological processes commonly rely on inorganic ammonia, ammonium salts, urea, or organic substrates like peptone or yeast extract as nitrogen sources because they support fastest growth (Reitzer 1996). In contrast to the extensive engineering efforts to broaden the carbon source spectrum for major biotechnological workhorses (Wendisch et al. 2016), only few alternative, non-conventional nitrogen sources have been made accessible. However, their utilization often is unfavorable due, e.g., to the carcinogenicity of melamine (Shaw et al. 2016). Formamide by contrast shows low toxicity (Kennedy 2014), and its presence in concentration of up to 160 and 700 mM only had a minor inhibitory effect on growth of *E. coli* and *C. glutamicum*, respectively (Ou et al. 2019; Schwardmann et al. 2023b). Moreover, the use of formamide does not suffer from nitrogen repression if supplemented as the sole source of nitrogen, but not as the primary carbon source (Guo et al. 2020a; Schwardmann et al. 2023b). Both studies recruited formamidase AmiF from *H. pylori* (Van Vliet et al. 2003; Guo et al. 2020a; Schwardmann et al. 2023b), whereas the anticontamination system for *E. coli* relied on AmiF from *P. pasadenensis* (Guo et al. 2017; Ou et al. 2019), that only share 48% identity of their amino acid sequences.

In the context of exploiting the formamidase/formamide strategy as anticontamination system, non-sterile fermentation of a *B. subtilis* acetoin producer strain, supplemented with 60 mM formamide as sole source of nitrogen, yielded a titer of about 25 g L^−1^ (Table 2), which is comparable to those from conventional media (Guo et al. 2020a). As acetoin does not contain nitrogen atoms, nitrogen from formamide was exclusively allocated to the synthesis of biomass and natural metabolites but did not end up in the product. Thus, restricted nitrogen availability did not limit production. The growth limitation upon nitrogen deprivation provides a potential tool to perform a two-stage cultivation with growth-accompanied production during the first phase until nitrogen is depleted, followed by sole product synthesis in the second phase. This concept of nutrient content–dependent two-stage cultivation was successfully used to decouple amino acid production from growth in *C. glutamicum* strains, engineered to prevent glucose utilization for growth, allocating it exclusively to production after acetate depletion (Blombach et al. 2008). Similarly, it could be elaborated for formamide to completely decouple biomass and biosynthesis of nitrogen-free products. For the bioconversion of xylose to produce xylitol and xylonate in *C. glutamicum* (Schwardmann et al. 2023a)*,* nitrogen starvation–inducible promoters combined with balanced provision of ammonia allowed for growth-decoupled production. What these approaches have in common is that they only work for non-nitrogenous target compounds such as acetoin or xylitol. Notably, nitrogen limitation has often been used to improve polyhydroxybutyrate production by strains of *C. necator* due to the increased NADPH availability (Zhang et al. 2022). Therefore, the implementation of the formamide/formamidase system as a selective trait in this organism may be interesting for polyhydroxybutyrate production under non-sterile conditions in a two-stage process.

**Table 2:** Formamide-based enzyme expression and production by engineered strains of *E. coli*, *B. subtilis*, and *C. glutamicum*, overexpressing formamidase genes, with formamide as a sole source of nitrogen with maximal reported titers or the method used for verification of formamidase activity

Product	Organism	Formamidase gene	Formamide (mM)	Glucose (g L^−1^)	Cultivation system	Product titer (g L^−1^)/method of verification	Reference
Gfp	*E. coli* BL21(DE3)	*H. pylori* 26695	48	7	Shake-flask, non-sterile	Fluorescence microscopy	(Ou et al. 2019)
Chitinase	*E. coli* BL21(DE3)	*H. pylori* 26695	48	7	Shake-flask, non-sterile	Chitin hydrolysis	(Ou et al. 2019)
Acetoin	*B. subtilis*	*P. pasadenensis* CS0611	60	68	Fed-batch, non-sterile	25.65	(Guo et al. 2020a)
L-glutamate	*C. glutamicum*	*H. pylori* 26695	60	20	Shake flask	6.51	(Schwardmann et al. 2023b)
L-lysine	*C. glutamicum*	*H. pylori* 26695	60	20	Shake flask	5.65	(Schwardmann et al. 2023b)
*N*-methyl-phenylalanine	*C. glutamicum*	*H. pylori* 26695	60	20	Shake flask	1.68	(Schwardmann et al. 2023b)
Dipicolinic acid	*C. glutamicum*	*H. pylori* 26695	60	20	Shake flask	0.56	(Schwardmann et al. 2023b)

**Fig. 4:**
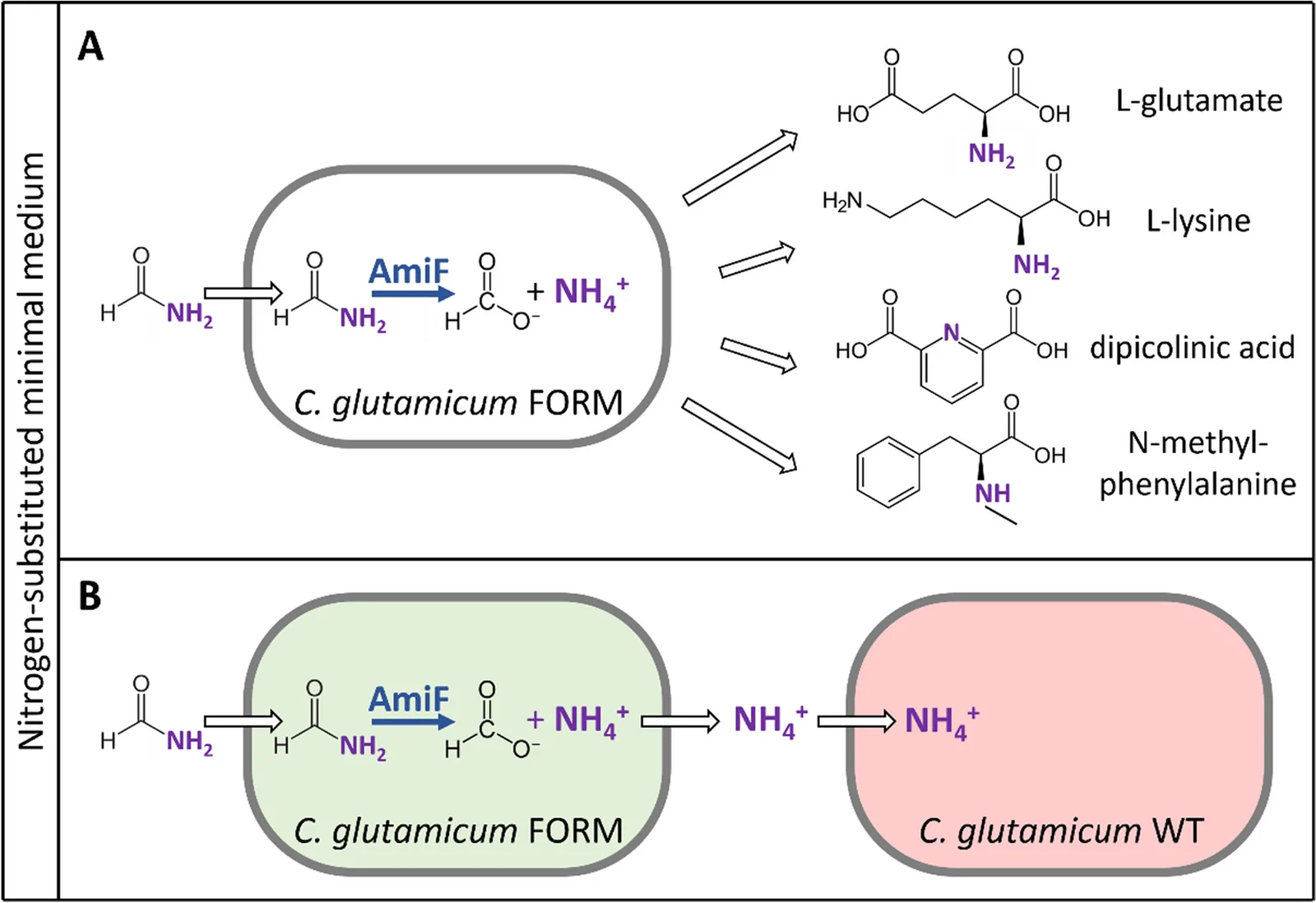
Engineering of *C. glutamicum* for formamide-based production of nitrogenous compounds (**A**) and for co-cultivation with a formamidase-deficient *C. glutamicum* strain (**B**). *C. glutamicum* was engineered to overexpress formamidase (AmiF) gene from *H. pylori* 26695 (FORM, blue) to produce L-glutamate, L-lysine, dipicolinic acid, and *N*-methylphenylalanine in nitrogen-substituted minimal salt medium using glucose and formamide as sole sources of carbon and nitrogen. Ammonium release from formamide hydrolysis by AmiF by a formamidase-positive *C. glutamicum* strain (FORM) supports growth of a formamidase-deficient *C. glutamicum* strain (WT) in co-cultivation. For differentiation, strains were overexpressing genes either for the fluorescence protein Gfp_UV_ (green) or Crimson (red) (**B**)

To target formamide-based production of nitrogenous compounds such as amines and amino acids, high concentrations of formamide have to be supplemented as nitrogen source for growth and production. This has been achieved for *C. glutamicum* (Schwardmann et al. 2023b). Stable isotope labeling using ^15^N-labeled formamide or ammonium sulfate confirmed the incorporation of nitrogen from formamide into both biomass and the exemplary nitrogenous product L-lysine (Schwardmann et al. 2023b). Notably, the simultaneous provision with natively accessible ammonium sulfate and xenobiotic formamide revealed similar acceptance and incorporation of nitrogen from both substrates into biomass (Schwardmann et al. 2023b). Beyond formamide-based production of the feed amino acid L-lysine, the system was transferred to established producer strains for formamide-based production of the food amino acid L-glutamate, the *N*-alkylated amino acid *N*-methylphenylalanine, and the aromatic dicarboxylate dipicolinic acid (Schwardmann et al. 2023b) (Fig. 4A, Table 2). Formamide was even superior to the standard nitrogen source mixture of urea and ammonium sulfate for yielding up to 80% increased titers of all four products (Schwardmann et al. 2023b).

Formamide-based production using recombinant *E. coli* and *C. glutamicum* strains revealed a clear tradeoff between immediate fast growth at low formamide concentrations and the support of higher biomass formation with increasing formamide concentrations (Guo et al. 2020a; Schwardmann et al. 2023b). Accumulation of formate as a degradation product from formamide hydrolysis was detected, and its growth inhibitory effect (Witthoff et al. 2012; Schwardmann et al. 2023b) limited cultivation at higher formamide concentrations. The formate problem was solved by oxidation to carbon dioxide. To this end, either native NAD-dependent formate dehydrogenase (Witthoff et al. 2012) or heterologous NADP-dependent formate dehydrogenase variant from *Pseudomonas* sp. 101 (Calzadiaz-Ramirez et al. 2020) was used (Schwardmann et al. 2023b). Probably reflecting the catabolic nature of formamide utilization as nitrogen source, NAD-dependent formate dehydrogenase was superior to improve growth characteristics when higher formamide concentrations were used as nitrogen source (Schwardmann et al. 2023b).

A completely different application of formamide offers its use in synthetic microbial consortia, since these typically depend on the (mutual inter-)dependency of both microbial partner strains, e.g., based on cross-feeding of essential nutrients (Sgobba and Wendisch 2020). In nature, ammonium cross-feeding occurs between algae and N_2_-fixing or methylamine-degrading bacteria (Suleiman et al. 2016; Zecher et al. 2020; Ambrosio and Curatti 2021). In contrast to the exploitation of formamidase-driven formamide degradation as selective trait in *E. coli* and *B. subtilis* (Ou et al. 2019; Guo et al. 2020a), formamidase overexpression in *C. glutamicum* led to ammonium leakage into the medium. The leaked ammonium was sufficient to support growth of a second formamidase-deficient strain in co-cultivation with formamide as the sole nitrogen source (Schwardmann et al. 2023b). Differentiation between formamidase-positive and -deficient strains by the expression of either of the genes for the fluorescence proteins Gfp_UV_ and Crimson revealed that inoculation with about 10% formamidase-positive cells and 90% formamidase-negative cells was sufficient to support growth of both strains (Schwardmann et al. 2023b).

The demonstrated strict obligatory intra-species dependency (Fig. 4B) provides a promising basis for using nitrogen cross-feeding from formamide in combination with a second conversely cross-fed metabolite. This could be achieved in inter- or intra-species consortia. However, while these kinds of synthetic consortia are intensely discussed for potential application in biotechnology (McCarty and Ledesma-Amaro 2019; Sgobba and Wendisch 2020; Cao et al. 2022), a number of limitations have to be considered. Often, the metabolic costs of transport processes are neglected although primary or secondary active transport entails a metabolic burden (or cost in the form of ATP, ion gradients, etc.) and uptake/export of charged/uncharged species of a molecule imparts cost in form of the decoupled transmembrane pH/ion gradient perturbing ATP synthesis, e.g., by F_o_F_1_ ATP synthase (Krämer 1994). However, the metabolic cost associated with transport processes may be used in the design of synthetic microbial consortia by transport engineering (Pérez-García and Wendisch 2018). Thus, the demonstrated strict obligatory intra-species dependency via formamide/formamidase (Fig. 4B) is an interesting design option for synthetic consortia, but it has to be kept in mind that division of labor in a synthetic consortium must outcompete monocultures that perform all labor by one cell to achieve applicability in biotechnology.

## Outlook

The utilization of formamide/formamidase shows application potential for non-sterile fermentations, as rare nitrogen source for fermentative production under growth and non-growth conditions, as well as in synthetic microbial consortia. However, some inherent limitations characterize the formamide/formamidase system. Although formamide shows low toxicity towards cells as it affects DNA and RNA helicity (Blake and Delcourt 1996), it inhibits bacterial oxidases (Gupta and Mazumdar 2008) and dissociates and inactivates some enzymes such as *E. coli* alkaline phosphatase (Falk et al. 1982). For applications in thermophiles, additional considerations have to be taken since hot formamide introduces formyl groups into proteins to generate formyl-glycyl and diformyl-lysyl residues (Perkins 1965). Addition of amino acids and/or purines to the growth medium may alleviate some toxic effects as shown for *E. coli* (Wheeler and Grammer 1960).

Inhibition due to formate generated from formamide by formamidase was overcome by the overexpression of formate dehydrogenase genes in engineered bacteria. This mimics the fast assimilation or dissimilation of formate in natural formamidase-positive bacteria. However, since some formamidase enzymes show slower hydrolysis of other short-chain amides such as acetamide, propanamide, and butanamide, these side reactions may perturb growth and production with formamide (Clarke 1969; Wyborn et al. 1996; Soriano-Maldonado et al. 2011).

The enzymatic activity of formamidase may be relevant in cell-free biocatalaysis or whole-cell biotransformation. Purified *B. cereus* formamidase was used in cross-linked enzyme crystals (CLECs) in a microfluidic setup (Conejero-Muriel et al. 2015). Here, its side activity as acyl-transferase was used to produce acetohydroxamic acid, an inhibitor of urease used to treat chronic urea-splitting urinary infection (Lithostat®). It may be possible to use *B. cereus* formamidase in whole-cell biotransformation under non-growth conditions provided that acetohydroxamic acid may be exported by the producing cells. Another enzyme application may be similar to the use of the phosphite dehydrogenase/phosphite system. Fusions between the NADPH-dependent monooxygenase P450_BM3_ with phosphite dehydrogenase accepted phosphite as cheap electron donor for monooxygenase reactions (Beyer et al. 2017). In principle, it is conceivable to use formamidase to provide ammonia in situ for reactions of ammonia-dependent enzymes. In this respect, urease has been used to provide ammonia via hydrolysis of urea in situ for the synthesis of polyhydroquinoline and polyhydroacridine derivatives. The reactions of the aryl aldehydes with dimedone or ethyl acetoacetate and the in situ generated ammonia occurred in water under mild green conditions (Zhu and Li 2021). Besides ammonia, urease generates carbon dioxide gas but formamidase formic acid; thus, the application of these enzymes in bio-chemo-catalysis has to consider the respective by-products with regard to possible perturbation or beneficial aspects.

While first steps to exploit the formamide/formamidase system in fermentative production and enzyme catalysis, for non-sterile fermentations, and to tune synthetic microbial consortia have been taken, the full potential has yet to be tapped by future research.

## **Supplementary information**

ESM 1 The online version contains supplementary material available at ## to be entered after revision ##. (PDF 672 kb)

## Data Availability

All data are present in the manuscript and its Supplement.
