# Supplementary material for: Prospects of formamide as nitrogen source in biotechnological production processes
Source: Appl Microbiol Biotechnol. 2024 Jan 10;108:105. doi: 10.1007/s00253-023-12962-x (PMC10781810; doi:10.1007/s00253-023-12962-x)
Supplement: Supplementary file 1 [file 253_2023_12962_MOESM1_ESM.pdf]

Prospects of formamide as nitrogen source in biotechnological production processes

Lynn Schwardmann[1,#], Leonie Benninghaus[1], Steffen N. Lindner[2], Volker F. Wendisch[1*]

[1] Genetics of Prokaryotes, Faculty of Biology and CeBiTec, Bielefeld University, Universitätsstr. 25, 33615 Bielefeld, Germany

[2] Department of Biochemistry, Charite Universitätsmedizin Berlin, corporate member of Freie Universität Berlin and Humboldt-Universität

# current address: Aminoverse B.V., Daelderweg 9, 6361HK Nuth, the Netherlands

* address correspondence to Volker F. Wendisch volker.wendisch@uni-bielefeld.de

Tab. #S1 List of abbreviations used in Fig. #3 with identifiers, enzyme names, organisms, and amino acid sequence identity similarities. *n.s.s. = no significant similarity detectable by BLAST alignment.

Label	Identifier	Enzyme	Organism	Amino acid similarity [%]
AERPE	TR:Q9YEQ1	Formamidase	*Aeropyrum pernix* (strain ATCC 700893 / DSM 11879 / JCM 9820 / NBRC 100138 / K1)	*n.s.s.
AGRSK	SP:P60327	*N*-carbamoyl-D-amino acid hydrolase	*Agrobacterium* sp. (strain NK712)	24
ALCBS	SP:Q0VN20	Aliphatic amidase	*Alcanivorax borkumensis* (strain ATCC 700651 / DSM 11573 / NCIMB 13689 / SK2)	36
ALLAM	SP:B9K1J4	Formamidase	*Allorhizobium ampelinum* (strain ATCC BAA-846 / DSM 112012 / S4)	45
ARATH1	SP:Q8H183	Beta-ureidopropionase	*Arabidopsis thaliana*	28
ARATH2	SP:Q8VYF5	*N*-carbamoylputrescine amidase	*Arabidopsis thaliana*	28
ARATH3	SP:Q8VYF5-1	*N*-carbamoylputrescine amidase	*Arabidopsis thaliana*	27
ARATH4	SP:P46010	Nitrilase 3	*Arabidopsis thaliana*	34
ARATH5	SP:P32962	Nitrilase 2	*Arabidopsis thaliana*	32
ARATH6	SP:P32961	Nitrilase 1	*Arabidopsis thaliana*	30
ARATH7	SP:P32961-2	Nitrilase 1	*Arabidopsis thaliana*	30
ARATH8	SP:P46011	Bifunctional nitrilase/nitrile hydratase NIT4	*Arabidopsis thaliana*	28
BACAA	SP:C3P6U6	Formamidase	*Bacillus anthracis* (strain A0248)	77
BACAH	SP:A0RHV8	Formamidase	*Bacillus thuringiensis*	77
BACAN	SP:P59700	Formamidase	*Bacillus anthracis*	77
BACC0	SP:B7JK27	Formamidase	*Bacillus cereus* (strain AH820)	77
BACC2	SP:B7IVH6	Formamidase	*Bacillus cereus* (strain G9842)	77
BACC3	SP:C1EPV3	Formamidase	*Bacillus cereus* (strain 03BB102)	77
BACC4	SP:B7H6S5	Formamidase	*Bacillus cereus* (strain B4264)	77
BACCQ	SP:B9IW18	Formamidase	*Bacillus cereus* (strain Q1)	77
BACCR	SP:P59701	Formamidase	*Bacillus cereus* (strain ATCC 14579 / DSM 31 / CCUG 7414 / JCM 2152 / NBRC 15305 / NCIMB 9373 / NCTC 2599 / NRRL B-3711)	77
BACCZ	SP:Q635Y7	Formamidase	*Bacillus cereus* (strain ZK / E33L)	77
BACHK	SP:Q6HEM5	Formamidase	*Bacillus thuringiensis* subsp. *konkukian* (strain 97-27)	77
BACSP	SP:Q9L543	Aliphatic amidase	*Bacillus* sp.	37
BACSU	SP:P54608	Hydrolase YhcX	*Bacillus subtilis (strain 168)*	22
BOVIN	SP:Q2T9R6	Omega-amidase NIT2	*Bos taurus*	24
BRADU1	SP:Q89H51	Formamidase	*Bradyrhizobium diazoefficiens* (strain JCM 10833 / BCRC 13528 / IAM 13628 / NBRC 14792 / USDA 110)	44

BRADU2	SP:Q89VS2	Aliphatic amidase	*Bradyrhizobium diazoefficiens* (strain JCM 10833 / BCRC 13528 / IAM 13628 / NBRC 14792 / USDA 110)	39
BRASB	SP:A5EDA7	Aliphatic amidase	*Bradyrhizobium* sp. (strain BTAi1 / ATCC BAA-1182)	38
BRASO1	SP:A4Z3G9	Formamidase	*Bradyrhizobium* sp. (strain ORS 278)	46
BRASO2	SP:A4Z057	Aliphatic amidase	*Bradyrhizobium* sp. (strain ORS 278)	38
BURCH	SP:A0B137	Aliphatic amidase	*rkholderia cenocepacia* (strain HI2424)	37
BURO0	SP:B1K2X7	Aliphatic amidase	*Burkholderia orbicola*	36
BURO1	SP:Q1BP24	Aliphatic amidase	*Burkholderia orbicola* (strain AU 1054)	37
CUPNE	TR:A0A7T4FZZ8	Formamidase	*Cupriavidus necator*	46
DANRE	SP:Q4VBV9	Omega-amidase NIT2	*Danio rerio*	25
DELAS	SP:A9C011	Aliphatic amidase	*Delftia acidovorans* (strain DSM 14801 / SPH-1)	38
DESVH	SP:Q72CW9	Formamidase	*Desulfovibrio vulgaris* (strain ATCC 29579 / DSM 644 / NCIMB 8303 / VKM B-1760 / Hildenborough)	74
DESVV	SP:A1VEP0	Formamidase	*Desulfovibrio vulgaris* subsp. *vulgaris* (strain DP4)	74
DICDI	SP:Q557J5	Deaminated glutathione amidase	*Dictyostelium discoideum*	24
EMEND	TR:Q9C453	Formamidase	*Emericella nidulans*	*n.s.s.
ENSAD	SP:Q5S260	*N*-carbamoyl-D-amino acid hydrolase	*Ensifer adhaerens*	24
FRATT	SP:Q5NHL7	Citrullinase	*Francisella tularensis* subsp. *tularensis* (strain SCHU S4 / Schu 4)	24
GEOSE	SP:Q9RQ17	Aliphatic amidase	*Geobacillus stearothermophilus*	38
HELAH1	SP:Q17WY3	Formamidase	*Helicobacter acinonychis* (strain Sheeba)	99
HELAH2	SP:Q17YA2	Aliphatic amidase	*Helicobacter acinonychis* (strain Sheeba)	37
HELP21	SP:B6JN78	Formamidase	*Helicobacter pylori* (strain P12)	99
HELP22	SP:B6JKM3	Aliphatic amidase	*Helicobacter pylori* (strain P12)	37
HELPG1	SP:B5Z8N3	Formamidase	*Helicobacter pylori* (strain G27)	99
HELPG2	SP:B5ZA60	Aliphatic amidase	*Helicobacter pylori* (strain G27)	37
HELPH1	SP:Q1CS25	Formamidase	*Helicobacter pylori* (strain HPAG1)	99
HELPH2	SP:Q1CUK9	Aliphatic amidase	*Helicobacter pylori* (strain HPAG1)	37
HELPJ1	SP:Q9ZJY8	Formamidase	*Helicobacter pylori* (strain J99 / ATCC 700824)	99
HELPJ2	SP:Q9ZME1	Aliphatic amidase	*Helicobacter pylori* (strain J99/ ATCC 700824)	37
HELPS1	SP:B2UV01	Formamidase	*Helicobacter pylori* (strain Shi470)	99
HELPS2	SP:B2USC5	Aliphatic amidase	*Helicobacter pylori* (strain Shi470)	37
HELPY1	SP:O25836	Formamidase	*Helicobacter pylori* (strain ATCC 700392 / 26695)	100
HELPY2	SP:O25067	Aliphatic amidase	*Helicobacter pylori* (strain 700392 / 26695)	37
HUMAN	SP:Q9UBR1	Beta-ureidopropionase	*Homo sapiens*	25
LUPAN1	SP:Q3LRV4	Bifunctional nitrilase/nitrile hydratase NIT4A	*Lupinus angustifolius*	28

LUPAN2	SP:Q5QGZ8	Bifunctional nitrilase/nitrile hydratase NIT4A	*Lupinus angustifolius*	30
METME	SP:Q50228	Formamidase	*Methylophilus methylotrophus*	*n.s.s.
MOUSE1	SP:Q8VDK1	Deaminated glutathione amidase	*Mus musculus*	22
MOUSE2	SP:Q8VDK1-2	Isoform 2 of Deaminated glutathione amidase	*Mus musculus*	22
MOUSE3	SP:Q9JHW2	Omega-amidase NIT2	*Mus musculus*	24
MYCSM	SP:Q50228	Formamidase	*Methylophilus methylotrophus*	*n.s.s.
NOCFA	SP:Q5Z1U0	Aliphatic amidase	*Nocardia farcinica* (strain IFM 10152)	39
ORYSJ1	SP:Q93XI4	*N*-carbamoylputrescine amidase	*Oryza sativa* subsp. *japonica*	28
ORYSJ2	SP:Q6H849	Bifunctional nitrilase/nitrile hydratase NIT4	*Oryza sativa* subsp. *japonica*	31
PAENI	SP:Q93NG1	2-oxoglutaramate amidase	*Paenarthrobacter nicotinovorans*	23
PARBR	TR:A0A1E2XT43	Formamidase	*Paracoccidioides brasiliensis*	*n.s.s.
PHOLL	SP:Q7N278	Formamidase	*Photorhabdus laumondii* subsp. *laumondii* (strain DSM 15139 / CIP 105565 / TT01)	65
PONAB	SP:Q5R4L6	Omega-amidase NIT2	*Pongo abelii*	25
PSEA7	SP:A6V262	Aliphatic amidase	*Pseudomonas aeruginosa* (strain PA7)	37
PSEA8	SP:B7V2X1	Aliphatic amidase	*Pseudomonas aeruginosa* (strain LESB58)	37
PSEAB	SP:Q02QK0	Aliphatic amidase	*Pseudomonas aeruginosa* (strainUCBPP-PA14)	37
PSEAE	SP:P11436	Aliphatic amidase	*Pseudomonas aeruginosa* (strain / 1C / PRS 101 / PAO1)	37
PSEFS	SP:C3K9E6	Aliphatic amidase	*Pseudomonas fluorescens* (strain SBW25)	36
PSEP1	SP:A5W2C0	Aliphatic amidase	*Pseudomonas putida* (strain ATCC 700007 / DSM 6899 / BCRC 17059 / F1)	39
PSEPH	SP:P55176	Hydrolase in *pqqF* 5'region	*Pseudomonas protegens* (strain DSM 19095 / LMG 27888 / CFBP 6595 / CHA0)	24
PSESM	SP:Q887D9	Formamidase	*Pseudomonas syringae* pv. *tomato* (strain ATCC BAA-871 / DC3000)	44
PSESP	SP:Q75SP7	(R)-stereoselective amidase	*Pseudomonas* sp.	26
PSEU2	SP:Q4ZXA2	Formamidase	*Pseudomonas syringae* pv. *syringae* (strain B728a)	45
PYRAB	SP:Q9UYV8	Nitrilase	*Pyrococcus abyssi* (strain GE5 / Orsay)	27
RAT1	SP:Q03248	Beta-ureidopropionase	*Rattus norvegicus*	23
RAT2	SP:Q497B0	Omega-amidase NIT2	*Rattus norvegicus*	23
RHIFH	SP:G9AIU0	Aliphatic nitrilase	*Rhizobium fredii* (strain HH103)	29
RHIML	TR:H0G2A6	Formamidase	*Sinorhizobium meliloti* CCNWSX0020	*n.s.s.
RHIRD	SP:Q5S260	*N*-carbamoyl-D-amino acid hydrolase	*Ensifer adhaerens*	24
RHOE4	SP:C0ZNE8	Aliphatic amidase	*Rhodococcus erythropolis* (strain PR4 / NBRC 100887)	40
RHOER	SP:Q01360	Aliphatic amidase	*Rhodococcus erythropolis*	39

SCHPO1	SP:O59829	Probable nitrilase C965.09	*Schizosaccharomyces pombe* (strain 972 / ATCC 24843)	24
SCHPO2	SP:Q10166	Hydrolase C26A3.11	*Schizosaccharomyces pombe* (strain 972 / ATCC 24843)	24
SOLLC	SP:Q9XGI9	*N*-carbamoylputrescine amidase	*Solanum lycopersicum*	27
SOLTU	SP:Q3HVN1	*N*-carbamoylputrescine amidase	*Solanum tuberosum*	27
STAAU	SP:P55177	Hydrolase in *agr* operon	*Staphylococcus aureus*	25
STALU	SP:P55178	Hydrolase in *agr* operon (Fragment)	*Staphylococcus lugdunensis*	28
THISH	SP:B8GQ39	Aliphatic amidase	*Thioalkalivibrio sulfidiphilus* (strain HL-EbGR7)	38
TOBAC1	SP:Q42966	Bifunctional nitrilase/nitrile hydratase NIT4B	*Nicotiana tabacum*	33
TOBAC2	SP:Q42965	Bifunctional nitrilase/nitrile hydratase NIT4A	*Nicotiana tabacum*	33
UKNPR	SP:Q6RWK4	Nitrilase 2	Unknown prokaryotic organism	29
VARPS	SP:C5CWZ4	Aliphatic amidase	*Variovorax paradoxus* (strain S110)	38
XENLA1	SP:Q6IR61	Omega-amidase NIT2-A	*Xenopus laevis*	25
XENLA2	SP:Q6INI7	Omega-amidase NIT2-B	*Xenopus laevis*	26
XENTR	SP:Q28IE5	Omega-amidase NIT2	*Xenopus tropicalis*	26
YEAST	SP:P49954	Omega-amidase NIT3	*Saccharomyces cerevisiae* (strain ATCC 204508 / S288c)	24
